# Late-onset of immunodysregulation, polyendocrinopathy, enteropathy, x-linked syndrome (IPEX) with intractable diarrhea

**DOI:** 10.1186/s13052-014-0068-4

**Published:** 2014-10-18

**Authors:** Daniele Zama, Ilaria Cocchi, Riccardo Masetti, Fernando Specchia, Patrizia Alvisi, Eleonora Gambineri, Mario Lima, Andrea Pession

**Affiliations:** Pediatric Oncology and Haematology Unit “Lalla Seràgnoli”, Department of Pediatrics, University of Bologna Sant’Orsola-Malpighi Hospital, Via Massarenti, 11, Bologna, 40138 Italy; Pediatric Department, Maggiore Hospital, Bologna, Italy; Department of ‘NEUROFARBA’, Section of Child’s Health, University of Florence, Florence, Italy; BMT Unit, Department of Hematology-Oncology, Anna Meyer Children’s Hospital, Florence, Italy; Department of Pediatric Surgery, University of Bologna, Bologna, Italy

**Keywords:** Immunodysregulation polyendocrinopathy enteropathy X-linked syndrome (IPEX), Sirolimus, Forkhead box P3 (FOXP3)

## Abstract

The syndrome of immune dysregulation, polyendocrinopathy, enteropathy, X linked (IPEX) is a rare disorder caused by mutations in the FOXP3 gene. Diarrhea, diabetes and dermatitis are the hallmark of the disease, with a typical onset within the first months of life. We describe the case of a twelve-year old male affected by a very late-onset IPEX with intractable enteropathy, which markedly improved after starting Sirolimus as second-line treatment. This case suggests that IPEX should always be considered in the differential diagnosis of watery intractable diarrhea, despite its unusual onset.

## Correspondence

The syndrome of immune dysregulation, polyendocrinopathy, enteropathy, X-linked (IPEX) is a rare disorder, characterized by diarrhea, type-1 diabetes mellitus (T1DM) and dermatitis with onset within the first months of life [1,2]. Diarrhea is intractable and persists despite dietary exclusions and bowel rest, resulting in malabsorption and failure to thrive [3]; T1DM can precede or follow enteritis [4-6]; dermatitis is severe with eczematiform, ichthyosiform or psoriasiform aspects [7-10], other autoimmune diseases are often associated [11].

IPEX is caused by germ-line mutations in the FOXP3 gene, a key regulator of immune tolerance, located in the X-chromosome at Xp11.23-Xq13.3 [12-17]. It is critical for the function of CD4^+^CD25^+^ regulatory T-cells (T_REG_) and for the maintenance of peripheral immunologic tolerance [17,18].

### Findings

We describe a 12-year-old boy born at term from natural birth after an uncomplicated pregnancy from unrelated parents, referred to our hospital for severe enteritis started one month before with liquid mucus-haematic diarrhoea (height: 50^th^ centile, weight: 10^th^ centile, regularly vaccinated). No potentially triggering events have been reported, such as vaccinations, viral infections or changes in nutrition. In his past history he had recurring episodes of mild atopic dermatitis since the first year of life, a high level of total IgE (400 UI/L), and a constipated bowel (once every two/three days).

On admission, he was dehydrated (7% of weight loss). Blood tests revealed hypoproteinaemia and hypogammaglobulinemia (Table 1), so albumin was replaced.

**Table 1:** **The molecular and clinical features of the patient with IPEX who received sirolimus have been reported**

**Patient**		**Mutation**				**Clinical features**	**Histology**	**Management**			**Outcome**	**Ref.**
	**Age at onset age at dg**	**Nucleotide change**	**AA change**	**FOXP3**	**Molecular defect**			**Previous therapy**	**SIR**	**HSCT**		
1	7 y 10 y	c.968-20A>C	NA	NA	NA	Dermatitis, enteropathy	Lymphoplasmocellular eosinophilic infiltrate. Villous atrophy.	Steroids, AZA, CsA, FK, MTX. TPN, Total colectomy at 10 y	Y	N	Stable at 16 yr on SIR+MTX.	[19]
2^*^	2 m NA	NA				Enteropathy, erythematous eczema-like dermatitis	Lymphoplasmocellular infiltrate with marked eosinophilia. High rate of enterocyte apoptosis. Subtotal villous atrophy.	Steroids, FK, AZA	Y	N	Stable for 1.5 yr on SIR+AZA	[19]
3^*^	2 m NA	NA				Enteropathy, erythematous eczema-like dermatitis	Similar findings with that of his brother (pt.4)	Steroids, FK; AZA	Y	N	Stable for 6 m on SIR+AZA	[19]
4	2 y 4 y	1061 delC	Frameshift P354Q	NA	Premature stop codon. Truncated FKH domain	Enteropathy, nonspecific dermatitis	Mild villous blunting	Metronidazole, steroids, mesalamine, IFX, AZA, 6-MP	Y	N	Stable at 7 yr	[20]
5	1 w 7 y	200G>T	Q70H	NA	Predicted abnormal reading frame	Eczema, enteropathy, AHA, ITP, arthritis	Inflammation with villous atrophy	IVIG, steroids, TPN, antibiotics	Y	N	Stable at 8 yr	[20,21]
6^*^	3 w NA	g.-6247-4859del	NA	↓	Accumulation of unspliced mRNA	Skin/food allergies, Enteropathy, erythematous- eczematous skin rash	Lymphoplasmocellular infiltrate with marked eosinophilia. High rate of enterocytes apoptosis. Severe to total villous atrophy	Steroids, FK, AZA TPN	Y	N	Stable for 6 yr on SIR+AZA	[22]
7^*^	2 m NA	g.-6247-4859del	NA	↓	Accumulation of unspliced mRNA	Skin/food allergies, Eczema, Enteropathy	NA	Steroids, FK, AZA TPN	Y	N	Stable for 4 yr on SIR+AZA	[22]
8	5 w NA	g.-6247-4859del	NA	↓	Accumulation of unspliced mRNA	Enteropathy, Eczema, Allergy	NA	Steroids, FK, AZA	Y	N	Stable at 9 yr on SIR+AZA	[23]
9	3 w NA	g.-6247-4859del	NA	↓	Accumulation of unspliced mRNA	Enteropathy, Eczema, HP gastritis, Allergy	NA	Steroids, FK AZA	Y	N	Stable at 6 yr on SIR+AZA	[23]
10	Birth NA	g.-1121 T>G	F374C	↓	Full length FOXP3 with abnormal FKH domain	T1DM, HTH, Enteropathy, Eczema, AHA, ITP, Allergy.	NA	Steroids, FK506	Y	N	Died at 14 m during HSCT induction	[23]
11	6 w NA	751-753 del GAG	E251del	↓	Disrupts FOXP3 oligomerisation	Enteropathy, Eczema, HTH, Interstitial Nephritis, AHA, Allergy.	NA	FK506	Y	Y	Died at 10 yr after HSCT	[23]
12	1 m 6 y	1150G>A	A384T	↓	Full length FOXP3 with abnormal FKH domain	Enteropathy, Eczema, FTT, T1DM, AHA, Interstitial Pneumonia, Alopecia, Thyroiditis.	Eosinophil infiltration without villous atrophy	IVIG, CsA, steroids, TPN, fludarabine-autologous lymphocytes, FK, MTX, Rituximab, cyclophosphamide.	Y	N	Stable at 16 yr on others drugs	[4,24,25]
13	Birth 7 w	1150G>A	A384T	↓	Full length FOXP3 with abnormal FKH domain	Enteropathy, T1DM, Exfoliative Dermatitis, HTH, Pancytopenia	NA	TPN	Y	N	Died at 7 w	[26]
14	Birth 4½ y	AAUAAA/AAUAAG	NA	↓	Polyadenylation defect resulting in unstable FOXP3 mRNA	Enteropathy, Dermatitis, FTT, T1D.	NA	MTX, steroids, TPN.	Y	Y	Stable at 1 yr	[27]
15	1 w	1015C>G	P339A	↓	Missense mutation. Predicted to yield full length FOXP3	Enteropathy, Eczema, T1DM, FTT, Euthyroid Thyroiditis, AIH, AHA	Villous atrophy	Steroids, FK; AZA	Y	N	Died at 5.5 m before HSCT	[28]
16	3 m 1y	Exon 10	NA	NA	NA	FTT, Enteropathy, Eczematous Dermatitis, ITP stomatitis	NA	Cyclophosphamide, VCR, TPN	Y	N	Stable 2½ yr on other drugs	[29]

^*^Brothers; 6-MP 6-Mercaptopurina; AHA autoimmune haemolytic anaemia; AIH Autoimmune hepatits; AZA Azathioprine; CsA Cyclosposporine; FTT: failure to thrive; FK: tacrolimus; HSCT hematopoietic stem cell transplantation; HTH Hypothyroidism; IFX Infliximab; ITP immune thrombocytopenic purpura; IVIG Intravenous Immunoglobulin; Y: Yes; yr: years; m: months; MTX Methotrexate; NA Not Available; N: No; Ref. References; SIR Sirolimus; T1DM Type 1 Diabetes mellitus; TPN Total Parenteral Nutrition; VCR Vincristine; w: weeks; ↓: reduction of expression.

Abdominal ultrasound highlighted wall thickening of the bowel loops. Esophagogastroduodenoscopy (EGDS) and colonoscopy revealed ulcerative lesions at the stomach, duodenum, terminal ileum and colon, giving rise to a suspect of inflammatory bowel disease. Biopsies revealed villous blunting and inflammatory infiltration of the mucosa. After starting intravenous methylprednisolone, metronidazole and parenteral nutrition a partial remission was observed.

**Figure 1:**
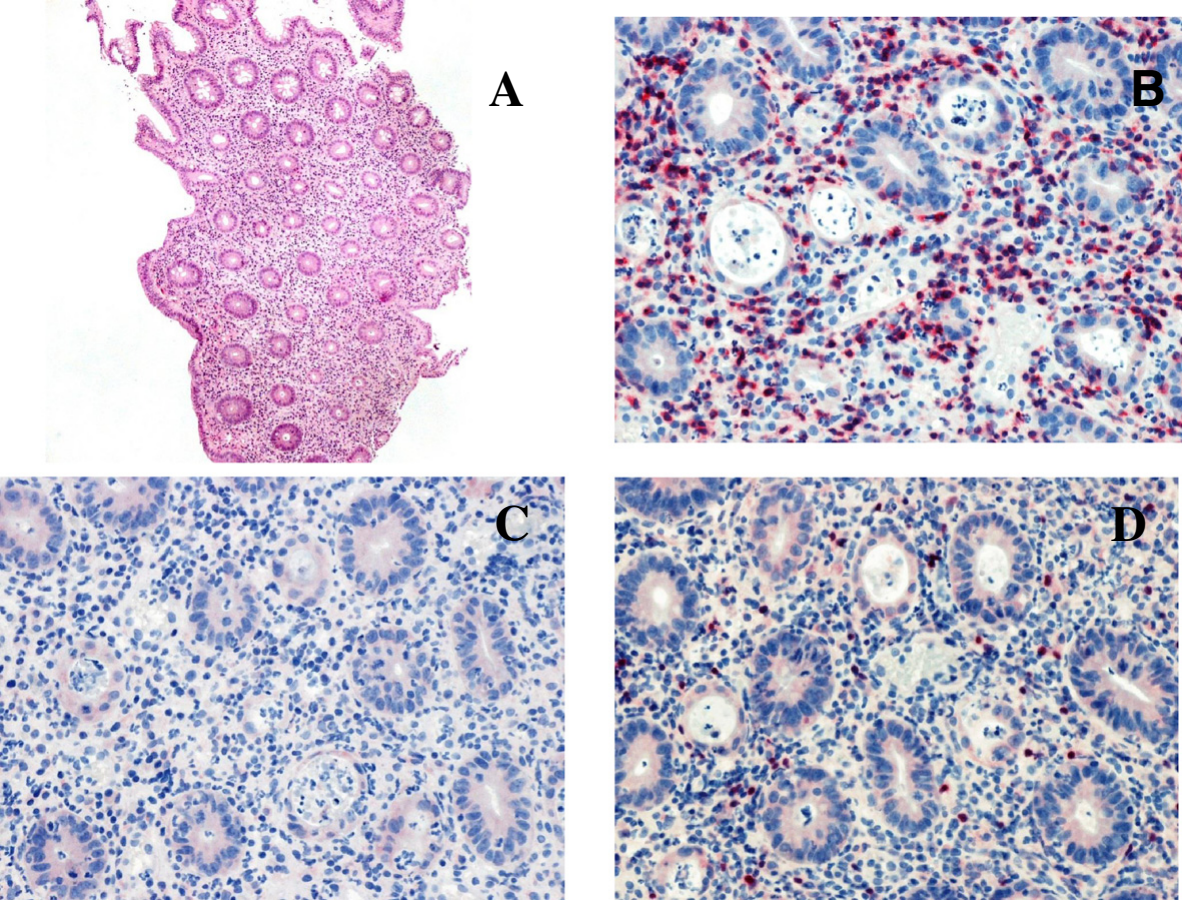
**Biopsy of the colon mucosa highlights a marked inflammatory infiltrate.** (**A**: H&E 5x) with a GVHD-like aspect characterized by a prevalence of lymphocytes CD8+ (red; **B**) than lymphocytes CD4+ (red; **C**). Rare lymphocytes expressing FOXP3 were found (red; **D**).

Ten days later, for a worsening of symptoms, EGDS and colonoscopy were repeated, with a superimposable picture. Particularly, the biopsies of the colon showed lympho-granulocytic acute inflammation with Graft versus Host Disease-like aspect, a lesion typically reported in IPEX (Figure 1) [30]. Due to the inability to control the symptoms the patient underwent ileostomy.

Despite the age of the patient was atypical for the onset of IPEX, we evaluated the presence of autoantibodies to harmonin, which resulted positive (>100 U.A.). Then, diagnosis was confirmed by the genetic examination of FOXP3 gene, revealing a mutation in the exon 9 (1040G > A), with substitution of Arginine to Histidine (R347H). The mother resulted negative. The total number of lymphocyte and lymphocyte subpopulations was normal, particularly T_REG_ were 5% of the total number.

Intravenous cyclosporine (range: 200-350 mg/dl) and methylprednisolone (2 mg/kg) were started, which reduced diarrhea and abdominal pain. After sixty days of parenteral nutrition the patient returned to oral feeding with the normalization of albumin levels (Table 1). Because of the onset of post-prandial hyperglycaemias, we excluded T1DM (Table 1) and glycaemia normalized after tapering steroid therapy. For a new worsening of the disease we introduced sirolimus (0.15 mg/kg/day; range: 8-12 mg/dl). The patient improved with a progressive reduction of intensity and frequency of abdominal pain and mucus emission. A new colonoscopy highlighted a marked decrease of the inflammation. After thirty-four days since the beginning of sirolimus, cyclosporine was suspended. After twelve months the patient is well, without recurrence of the disease.

### Conclusions

This case indicates that IPEX can have an atypical age of presentation. Thus, it should always be considered in the differential diagnosis of intractable diarrhea.

Four patients have been previously reported with IPEX with the same amino-acid substitution (R347H) found in our patient. The age of onset for all these subjects was within the first year of life and the first symptoms were recurrent ear infection, high IgE levels, T1DM, and gastritis. All had gastrointestinal symptoms with failure to thrive: two intractable diarrhea, two severe gastritis with mucosal atrophy or eosinophilic infiltration. Other symptoms were: coombs-negative haemolytic anaemia, food allergy, pancreatic exocrine failure, intractable hypertension, intestinal metaplasia, steatorrhea, and hypogammaglobulinemia. Patients received corticosteroid and calcineurin inhibitors. One patient died after allogeneic hematopoietic stem cell transplantation (HSCT) due to an infection.

Recently, evidence that patients with a severe form of IPEX may have circulating FOXP3^+^ T cells, as it is the case of our patient, which suggests that the cellular basis for the disease may be a result of a functional defect of Treg cells [1,26]. Mainly, R347H mutated-FOXP3 has been demonstrated as effective as wild-type-FOXP3 in converting normal T cell into Treg in vitro [31] and in maintaining the ability to suppress the production of cytokines, hallmark of Treg cells, conferring suppressive capacity on CD4^+^ T cells.

In 2005, three patients were successfully treated with sirolimus [19]. Since then, 16 patients received sirolimus and nine are in complete or partial remission (Table 2). Considering that sirolimus seems to be as effective as the calcineurin inhibitors, with less toxic effects, it can be considered as a valid therapeutic option for bringing these patients to HSCT in their best clinical condition.

**Table 2:** **Variables of our patient at the time of admission to our hospital, when he started the second line therapy with Sirolimus and after three months since the begging of this therapy**

**Variables**	**Reference range, age and sex-adjusted**	**Admission**	**Start SIROLIMUS**	**3 months after SIROLIMUS**
**White-cell count — per mm** ^**3**^	4.5 - 13.5	15.01	4.04	5.01
**Hemoglobin — g/dl**	11.5 - 14.5	16.3	11.7	11.5
**Hematocrit —%**	35 - 42	46.0	34.4	35.7
**Differential count —%**				
Neutrophils	40.0 - 74.0	89.6	51.2	48.0
Lymphocytes	19.0 - 48.0	6.6	30.3	38.0
Monocytes	3.0 - 9.0	2.4	13.5	7.6
Eosinophils	0.0 - 6.0	0.4	1.7	4.4
Basophils	0.0 - 1.5	0.3	1.1	0.7
**Platelet count — per mm** ^**3**^	250 - 550	522	247	273
**Glucose — mg/dl**	60 - 100	125	107	77
**Insulinemia — microU/mL**	7 - 24		6.8	
**C-peptide — ng/mL**	1.1 - 4.4		2.7	
**Islet cell autoantibodies**	Neg		Neg	Neg
**Glutamic acid decarboxylase— UI/ml**	<10 Neg		Neg	Neg
	>10 Pos			
**UREA — mg/dl**	15 - 50	72	40	18
**Creatinine — mg/dl**	0.5 - 1	0.91	0.54	0.35
**Uric Ac. — mg/dl**	2.2 - 6.6	8.6	5.2	3.4
**Total Colesterol — mg/dl**	130 - 204		121	
**TG — mg/dl**	31 - 108		40	
**HDL — mg/dl**	> 35		62	
**LDL — mg/dl**	< 170		50	
**Electrolytes — mmol/L**				
Sodium	136 - 146	128	139	142
Potassium	3.5 - 5.3	5.5	4.3	4.3
Chlorine	98 - 106	85	103	105
Calcium	8.8 - 10.8	9.6	9.3	9.2
**Phosphorus — mg/dl**	2.9 - 5.4	7.6	5	4.4
**Magnesium — mg/dl**	1.6 - 2.6	2.2	1.6	2.1
**Plasma Osmolarity — mOsm/L**	278 - 305	266		
**Protein — g/dl**				
Total	6,4 - 8.1	4.1	6.2	6.7
Albumin	3.5 - 5	2.4	4.2	4.3
γ –Globulin —%	11.1 - 18.8	10.5	11.4	13.4
**Bilirubin — mg/dl**				
Total	0.20 - 1.10	1.54	0.44	0.3
Direct/Indirect	0.00-0.30/< 0.80	0.48/1.06	0.21/0.23	0.1/0.2
**AST/ALT — U/L**	< 38/< 41	44/34	16/10	22/17
**Total Amylase — U/L**	30 - 100	50	60	
**Iron — μg/dl**	53 - 119		47	52
**U.I.B.C./T.I.B.C. — μg/dl**	110-330/250-400		300/347	273/325
**Ferritin — ng/mL**	7 - 140		22	16
**TSH — microU/mL**	0.6 - 6.3		1.93	1.02
**FT3 — pg/mL**	2.5 - 5.5		3.6	4.1
**FT4 — pg/mL**	9.0 - 17.0		20.7	12.9
**ATA — UI/mL**	< 115		23	16
**Anti TPO Ab — UI/mL**	< 34		12	13
**ESR — mm**	< 15	6	15	9
**CRP — mg/dl**	< 0.5	0.05	2.05	0.09
**Ab anti harmonine IgG — U.A.**	< 3.0 absent		>100	
	> 0.3 present			
**ANA**	< 1:80		< 1:80	
**AMA**	< 1:40		< 1:40	
**ENA**	< 0,7 Neg		Neg	
	0.7 - 1-0 Bl			
	> 1.0 Pos			

ALT Alanine aminotransferase, AMA Anti-mitochondrial antibodies, ANA Antinuclear antibodies, anti-TPO Ab Anti-ThyroidPeroxidase Antibodies, AST aspartate aminotransferase, ATA Anti-Thyroglobulin Antibodies, Bl Borderline, CRP C-reactive protein, ENA Extractable Nuclear Antigens, ESR erythrocyte sedimentation rate, FT3 Free Triiodothyronine, FT4 Free Thyroxine, HDL High-Density Lipoprotein, LDL Low-Density Lipoprotein, Neg Negative, Pos Positive, T.I.B.C. Total iron-binding capacity, TG triglycerides, TSH Thyroid-Stimulating Hormone, U.I.B.C. Unsaturated Iron Binding Capacity.

## Consent

Written informed consent was obtained from the parents of the patient for publication of this Case report and any accompanying images. A copy of the written consent is available for review by the Editor-in-Chief of this journal.

## Ethical approval

Internal ethical committee of Sant-Orsola approved the study.

## Abbreviations

IPEX: Syndrome of immune dysregulation, polyendocrinopathy, enteropathy, X linked
T1DM: Type-1 diabetes mellitus
EGDS: Esophagogastroduodenoscopy
FKH: Forkhead/winged helix domain
mTOR: Mammalian target of rapamycin
HSCT: Hematopoietic stem cell transplantation
